# Characterization of *Neowestiellopsis persica* A1387 (Hapalosiphonaceae) based on the *cpc*A, *psb*A, *rpo*C1, *nif*H and *nif*D gene sequences

**DOI:** 10.1186/s12862-024-02244-z

**Published:** 2024-05-06

**Authors:** Bahareh Nowruzi, Lenka Hutarova, Dominika Vešelenyiova, James S. Metcalf

**Affiliations:** 1 Department of Biotechnology, Science and Research Branch, Islamic Azad University, Daneshgah Blvd, Simon Bulivar Blvd, Tehran, Iran; 2 Institute of Biology and Biotechnology, Faculty of Natural Sciences, University of Ss. Cyril and Methodius in Trnava, Trnava, Slovakia; 3 Department of Biological Sciences, Bowling Green State University, Bowling Green, OH 43403 USA; 4 Brain Chemistry Labs, Box 3464, Jackson, WY 83001 USA

**Keywords:** Cyanobacteria, Phylogenetic analysis, *Neowestiellopsis persica, cpc*A

## Abstract

**Background:**

Complex descriptions of new strains of cyanobacteria appear very frequently. The main importance of these descriptions concerns potential new substances that they could synthesise, as well as their different properties as a result of their different ecological niches. The main gene used for these descriptions is 16 S with ITS or whole genome sequencing. *Neowestiellopsis persica* represents a unique example of the influence of ecology on morphological changes, with almost identical 16 S identity. Although our previously described *Neowestiellopsis persica* strain A1387 was characterized by 16 S analysis, we used different molecular markers to provide a way to separate strains of this genus that are closely related at the genetic level.

**Materials and methods:**

In order to conduct an in-depth study, several molecular markers, namely *psb*A, *rpo*C1, *nif*D, *nif*H and *cpc*A were sequenced and studied in *Neowestiellopsis persica* strain A1387.

**Results:**

The results of the phylogenetic analysis, based on *cpc*A, showed that the studied strain A 1387 falls into a separate clade than *N. persica*, indicating that this signature sequence could be a useful molecular marker for phylogenetic separation of similar strains isolated in the future.

**Conclusions:**

Analysis of strain A1387 based on gene differences confirmed that it is a *Neowestiellopsis* strain. The morphological changes observed in the previous study could be due to different ecological and cultivation conditions compared to the type species. At the same time, the sequences obtained have increased our understanding of this species and will help in the future to better identify strains belonging to the genus *Neowestiellopsis*.

**Supplementary Information:**

The online version contains supplementary material available at 10.1186/s12862-024-02244-z.

## Highlights

Identifying the *cpc*A gene as a useful molecular marker for species identification in the genus *Neowestiellopsis.*

Characterizing new sequences of genes *nif*H, *nif*D, *cpc*A, *psb*A in the genus *Neowestiellopsis.*

First in-depth study of *Neowestiellopsis persica* A1387 using functional genes.

## Introduction

Identification of the true-branched cyanobacteria/cyanoprokaryota, which traditionally belong to the Hapalosiphon/Stigonematales clade [1], is usually challenging. The strains belonging to this clade have unique morphological characters, which unfortunately are not sufficient for species identification [2]. The family Hapalosiphonaceae, to which the genus *Neowestiellopsis* belongs, is a monophyletic clade. However, some genera within it, such as *Westiellopsis, Fischerella* and *Hapalosiphon*, are considered to be polyphyletic [2].

The existence of polyphyletic genera leads to the need to use sufficient different molecular markers to study the closely related species. In some cases, the lack of resolution of traditional genetic markers, mainly the 16 S rRNA, can lead to the need to use several different genes to identify species belonging to these genera.

Various protein coding genetic sequences have been used for inferring phylogenies within cyanobacteria/ cyanoprokaryota (*rpo*C1, B, *gyr*B, *rbc*LX, *cpc*BA-IGS and 16–23 S ITS) [3–8].

In the past, the use of different molecular markers such as *rpo*C1, *nif*D, *nif*H, *cpc*A and *psb*Ahas helped to resolve the problem with closely related species. The *rpo*C1 gene, which encodes the β-subunit of RNA polymerase, is a more discriminating genetic marker between closely related species [9]. This marker was recently used in the study and description of the genus *Minunostoc* [7] and species *Neocylindrospermum variakineticum* [10] and *Dulcicalothrix alborzica* [11].

The *psb*Agene, an important functional gene, is part of the photosystem II reaction center and encodes photosynthetic D1 proteins [16]. Multiple copies of this gene can be found in cyanobacteria /cyanoprokaryota, such as *Synechococcus* sp. [17]. In Nostocales, this gene shows great variability and can be present at 1 to 11 copies [12]. Although this molecular marker is not often used for phylogenetic studies, it has been used in studies of species belonging to *Aliinostoc* [13, 14] and *Synechococcus* [15]. The main problem with using this gene as a molecular marker, compared to the results from 16 S rRNA, is the difference in primer specificity. Because of this, the results of community studies may not be comparable [16].

The molybdenum-dependent nitrogenase (*nif*) structural genes appear to have a single origin in cyanobacteria. The highly conserved genes *nif*D and *nif*H encode dinitrogenase reductase, a protein subunit of the nitrogenase complex involved in N_2_ fixation. They are thought to have been inherited from a common cyanobacterial ancestor [20]. A total of 16 *nif* genes have been identified in cyanobacteria, forming different operons (*nif*BSU, *nif*ENXW, *nif*HDKand *nif*VZT) [20, 21]. Common to all N_2_ fixers, they are useful for characterizing diazotrophic communities and differentiating cyanobacterial genera [4]. These molecular markers have been used in studies focusing on the genera *Desmonostoc* [16], *Nunduva, Kyrtuthrix* [17], *Crocosphaera, Rippkaea, Zehria* [18] and others. The *nif*D also provides a phylogenetic signal [23] and has been used to elucidate the evolutionary relationships among heterocyte -forming cyanobacteria [24]. It also proved useful in distinguishing between two genera of heterocyte-forming cyanobacteria, *Nostoc* and *Anabaena* [23], where *nif*H failed [25]. The phycocyanin-encoding operon has perhaps been used in the past to resolve cyanobacterial taxonomy [8]. For phylogenetic resolution, conserved coding regions such as *cpcB* and *cpcA* were used, while the closely related species were separated by the highly variable intergenic spacer region (IGS) [19, 20]. These molecular markers have also shown good resolution in distinguishing between freshwater biofilm- forming, planktonic and terrestrial cyanobacteria [20].

They have recently been used in taxonomic studies of the genera *Arthrospira* [21], *Microcystis* [22] along with the species *Compactonostoc shennongjiaensis* [7] and *Raphidiopsis curvispora* [12]. Both *cpc*Aand *nif*H appear to be more useful for strain discrimination than the commonly used 16 S rRNA gene, which shows low intrageneric variability in many cyanobacteria [7].

The genus *Neowestiellopsis*, originally described by Kabirnaj et al. [23], was isolated from dried rice fields in Mazandaran, Iran. This genus forms a separate clade when using 16 S rRNA as a molecular marker, which is further supported by the unique shape of folding of secondary structures from 16 to 23 S rRNA sequences. Based on these data, two species were described, *Neowestiellopsis persica* and *Neowestiellopsis bilateralis.*Nowruzi et al. [24] also identified strain A 1387 as belonging to *N*. *persica* with 100% homology to *N*. *persica* SA33. However, significant morphological differences could be identified between these two strains, such as different branching type, lack of biseriate development of filaments, larger cells, presence of akinetes and monocytes in *N*. *persica* A1387.

The aim of the present study was to extend the original description of *N.persica* strain A1387 by sequencing and analyzing the*cpc*A, *rpo*C1, *psb*A, *nif*H and *nif*D genes, to obtain a better understanding of strains belonging to the genus *Neowestiellopsis*.

## Materials and methods

### Cultivation of Neowestiellopsis persica A1387

*Neowestiellopsis persica* A1387(Hapalosiphonaceae)was purchased from the Cyanobacteria Culture Collection (CCC) and Alborz herbarium at the Science and Research Branch, Islamic Azad University, Tehran, with the accession number A1387. Purified Cultures were maintained in BG11 medium at 28 ± 2ºC with periodic shaking (twice a day). The culture room was illuminated with ca. 50–55 µmol photons m^− 2^ s^− 1^ with a photoperiod of 14:10 h light: dark cycle [24].

### Molecular and sequence analysis

Genomic DNA was isolated from 16 to 18 day-old log phase cultures using the Himedia Ultrasensitive Spin Purification Kit (MB505). The manufacturer’s instructions were followed, with the exception of an increased incubation time for the lysis solutions AL and C1, which were set to 60 and 20 min, respectively. DNA fragments within the following genes were amplified using the oligonucleotide primers and PCR reactions listed in Table 1: *nif*D, *nif*H, *psb*A, *rpo*C1and*cpc*A. PCR reactions were performed using Bio-Rad reagents with the following PCRconditions and procedure: 25 µl aliquots containing 10–20 ng DNA template, 0.5 µM of each primer, 1.5 mM MgCl_2_, 200 µM dNTPs and 1U/µl Taq DNA polymerase. The PCR profiles for the different genes were carried out according to Table 1. PCR products were checked by electrophoresis on 1% agarose gels (SeaPlaque® GTG®, Cambrex Corporation), using standard protocols. The products were purified directly using the Geneclean® Turbo kit (Qbiogene, MP Biomedicals) and sequenced using the BigDye® Terminator v3.1 cycle sequencing kit (Applied Biosystems, Life Technologies).

**Table 1:** Target genes and oligonucleotide primers used in this study

Target gene/ sequence	Sequence 5 × 3´	Thermal profile	Reference
*cpc*A-IGS	5’-GGCTGCTTGTTTACGCGACA-3’ 5’-CCAGTACCACCAGCAACTAA-3’	94˚C, 5 min 30 × (92˚C, 1 min; 55˚C, 1 min; 72˚C, 2 min) 72˚C, 6 min 4˚C, ∞	Neilan et al., 1997
*rpo*C1	5’TGGGGHGAAAGNACAYTNCCTAA-3’ 5’GCAAANCGTCCNCCATCYAAYTGBA-3’		Głowacka et al., 2011
*psb*A	psbA86F (5’-TTTATGTGGGTTGGTTCGG-3’) psbA980R4 (5’-TGAGCATTACGCTCGTGC-3’)	94˚C, 5 min 35 × (94˚C, 60 s; 56˚C, 60 s; 72˚C, 60 s) 72˚C, 10 min 4˚C, ∞	Junier et al., 2007
*nif*H	5’CGTAGGTTGCGACCCTAAGGCTGA-3’ 5’-GCATACATCGCCATCATTTCACC-3’		GabyandBuckley, 2012
*nif*D	F: 5′-TCCGKGGKGTDTCTCAGTC-3′ R: 5′-CGRCWGATRTAGTTCAT-3′		Roeselers et al., 2007

The partial sequences were compared with the ones available in the NCBI database (Jun, 2023) using BLASTn. The BLAST X tool (blast.ncbi.nlm.nih.gov/Blast.cgi) was used for *psb*A, *rpo*C1, *cpc*A, *nif*H and *nif*D genes. The sequences were annotated for the coding regions using the NCBI ORF Finder and the ExPASY proteomics server. Nucleotide similarities were computed using program SIAS (Sequence Identity and Similarity) [25]: SIAS: Sequence identities and similarities. Available at: http://imed.med.ucm.es/Tools/sias.html and using the PAM250 matrix.

### Nucleotide sequence accession numbers

Sequence data were deposited in the DNA Data Bank of Japan (DDBJ) with the accession numbers: OP698106 (*cpc*A-IGS), OP698107 (*nif*H), OP698108 (*psb*A), OP698109 (*rpo*C1), and OP698110 (*nif*D). Number of nucleotides and amino acidfor *nifD* (372 and 124),

*nifH* (369 and 123), *psbA* (666 and 222), *rpoC1* (591 and197) and *cpc*A-IGS (270 and 90) genes were submitted in DDBJ.

### Phylogenetic analysis

The*psb*A, *rpo*C1, *cpc*A, *nif*H and *nif*D genes sequences obtained in this study, as well as the best hit sequences (> 94% identity) retrieved from GenBank, were first aligned using MAFFT version 7 [26] with automatic settings for nucleotide sequences. All alignments were visualised using Jaiview [27, 28] and then the alignments were used to build maximum likelihood phylogenetic tree for the genes. For this, we used IQ-Tree version 2 [29, 30]. TIM2e + G4 + F, TIM2e + I + G4 + F, TVMe + I + G4 + F, TIM3 + F + G4 and TIM3 + F + I + G4 models were used as suggested (BIC criterion) after employing model test implemented in IQ-tree for *nif*D, *nif*H, *psb*A, *rpo*C1 and *cpc*A-IGS genes respectively. Tree robustness was estimated with the bootstrap value set to percentages using 1000. The program MrBayes version 3.2.7a [27, 28, 31] were used for calculation of phylogenetic tree for each gene, where the Bayesian inference were considered. The Markov chain Monte Carlo (MCMC) algorithm, using default parameters was run for 10 000 generations with 2 runs of four incrementally heated chains, starting from random trees and sampling every 10 generations. The first of 25% of the trees were discarded as burn-in and the remaining trees were used to construct a 50% majority rule consensus tree for each gene.

Resulting trees were visualized in ITOL [32], available online at https://itol.embl.de/standard bootstrap and 10,000 ultrafast bootstrap to evaluate branch supports [33].

## Results

**Fig. 1:**
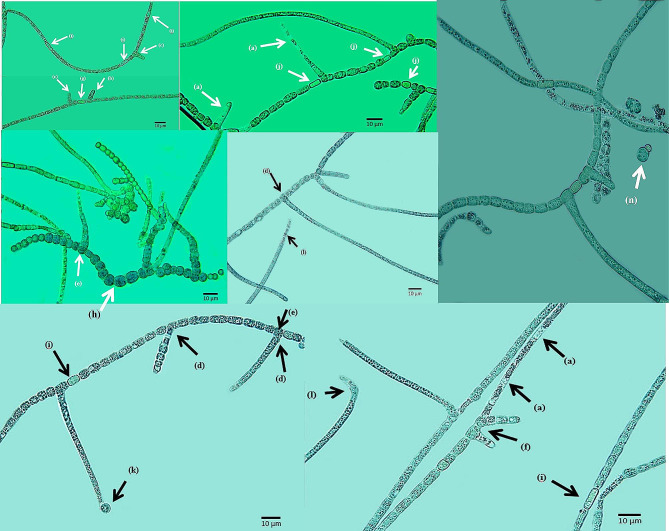
Morphological characterization of *NeowestiellopsisPersica* A1387. With increasing age there are significant increases in the number of main and branching filaments terminating in an empty sheath **(a).** Unilateral T-type branches arise from the main filament **(b)**; erect true branches (with T-type branching) **(c)** usually unilateral **(d).** Sometimes bilateral branching origins from one **(e)** or two near cells **(f)**. Sometimes two near cells are separated by a heterocyte **(g)**. Moreover, the studied strain may eventually differentiate a series of spherical, thick-walled cells that are akinetes **(h)**, 6.25 μm length × 3.75–5 μm width. Heterocytes are intercalary **(i)** and could be found near the branch **(j)**. Sometimes Hormogonia are formed at the end of a branch by one cell **(k)**, and also directly on the main trichomes **(l)**. Reproduction occurs via monocyte formation, which is a spherical cell, 3.5–5. 5 μm of diameter (**n**)

Our previous study, which focused on phylogenetic analyses based on 16 S rRNA sequences, suggested that this strain is genetically *N. persica*. However, a different morphology (Fig. 1), and the presence of genes responsible for the production of cyanotoxins indicated that this strain could be a different species [24].

When we compared the morphology of the studied strain with *Neowestiellopsis persica* SA33 (MF066912.1) and *N*. *bilateralis* SA16, we found differencesin morphological characterization (Table 2). The branching of *Neowestiellopsisbilateralis* was found on both sides of the main axis, however it occurred only on one side with our studied strain, more like *N*. *persica*. Our strain presented V and T type branching while *N. persica* and *N. bilateralis* only had T type branching. In *N*. *persica* SA33 biseriate development was observed, with terminal cells of branches tapered toward the apex and the first cell of the branch adjacent to main filament was irregular in shape, although these characteristics were never seen in our studied strain.

**Table 2:** Morphological observations of the studied strain. The latter was based on previously published photomicrographs

		Neowestiellopsis A1387	N. persica SA33	N. bilateralis SA16
**Thallus**		Creeping and erect filaments	The main filaments were thicker and creeping than the branches	The main filaments were thicker and creeping than the branches
**Heterotrichy/main** **axis/branches**		+/U & B/U	*+/*U &B/ U & B	+/U/U
**Color of Thallus**		olive green	greenish	bluish green
**Branching**		T-type only one side of main axis and V-type.	T-type only one side of main axis.	T-type both sides of main axis.
**Vegetative cells in main filaments**		spherical to rectangular, 0.7–1.1 × longer than wide, 6.5–13.5 μm length, 6.3–15.5 μm width	width usually much greater than length, 4.39–5.41 μm length, 7.52–9.29 μm width	square, cylindrical or barrel shape, 3.64–7.36 μm length, 4.8–10.62 μm width
**Vegetative cells in branching filaments**		spherical or slightly oblong, 3–8 × longer than wide, 11.2–29.5 μm length, 4.5–6.0 μm width	Irregular-shaped cells with some being squeezed from both sides, 6.13–6.19 μm length, 6.66–6.73 μm width	Irregular shaped cells with some being squeezed from both sides, 5.92–5.99 μm length, 6.33–6.44 μm width
**Heterocytes** **Inmainfilaments**		elongate, spherical, or even compressed (shorter than broad) intercalary 10.0–22.5 μm length × 6.5–11.5 μm width	Irregular shaped; Large cells and curved on the width,7.82–7.88 μm length, 10.82–10.89 μm width	Irregular shaped; Large cells and curved on the width, 8.00–8.09 μm length, 10.24–10.41 μm width
**Heterocytes** **Inbranchingfilaments**	**Tr**	7.3–8.0 μm length × 4.8–8.5 μm width	-	-
	**I**	5.3–6.0 μm length × 2.8–3.5 μm width	Large sized; always smaller than those of the main branches, 3.32–3.47 μm length, 4.30–4.38 μm width	Large sized; always smaller than those of the main branches, 6.92–6.95 μm length, 8.03–8.08 μm width
**Akinetes**		Oblong, mainly in chains, 5.0–6.0 μm broad, 6.5–11.0 μm length.	Not observed	Not observed
**Branching**		T and V	T	T
**Multiplication**		HG, A, Monocyte	HG	HG

^a^Type of thallus branching T - T-branching and V- V-branching), ^b^ HG- hormogonia; ^c^ A, akinetes), ^d^ heterotrichy that indicates differences in the shape of the cells of the main and secondary branches [+, clear differences; U, uniseriate; B, biseriate], ^e^ heterocyst position (Tr, terminal; I, intercalary)

In both *Neowestiellopsis* species, the main filament cells that gave rise to branches had irregular-shaped cells with some being squeezed from both sides, but there were no irregular-shaped cells in the studied strain and in total the mean size of vegetative cells, of both *N. bilateralis* and *N. persica*, were smaller than to the studied strain. However, the size of heterocytes in main and in branched filaments for both strains were in the same range.

In our strain, akinetes and monocyte reproductive cells were observed, but these were not reported for the other species of *Neowestiellopsis.*

In our present study, we focused more on the differences between *Neowestiellopsispersica* A1387and other strains belonging to the *Neowestiellopsis* cluster. Our analyses point out that available databases show a lack of sequences for genes other than *rpo*C1. For this reason, we decided to take a closer look at four more genes besides *rpo*C1, *psb*A, *nif*D, *nif*H, and*cpc*A. Concerning these genes, we identified the closest possible sequences from the same cluster as the original *Neowestiellopsis* strain, or from the strains from the closest clusters that belong to *Fischerella, Mastigocladus, Hapalosiphon* and *Westiellopsis.*

### Phylogenetic analyses

First, we created a multiple sequence alignment for each of the studied genes using MAFFT. Alignments were visualised as shown in Supplementary Figs. S1-S5 and similarities in the sequences were highlighted. Positions with the lowest level of sequence similarity are not highlighted, and positions showing the highest level of conservation are highlighted in dark blue. Consensus nucleotides for each position in the sequences are shown on the bottom of the multiple sequence alignment.

**Fig. 2:**
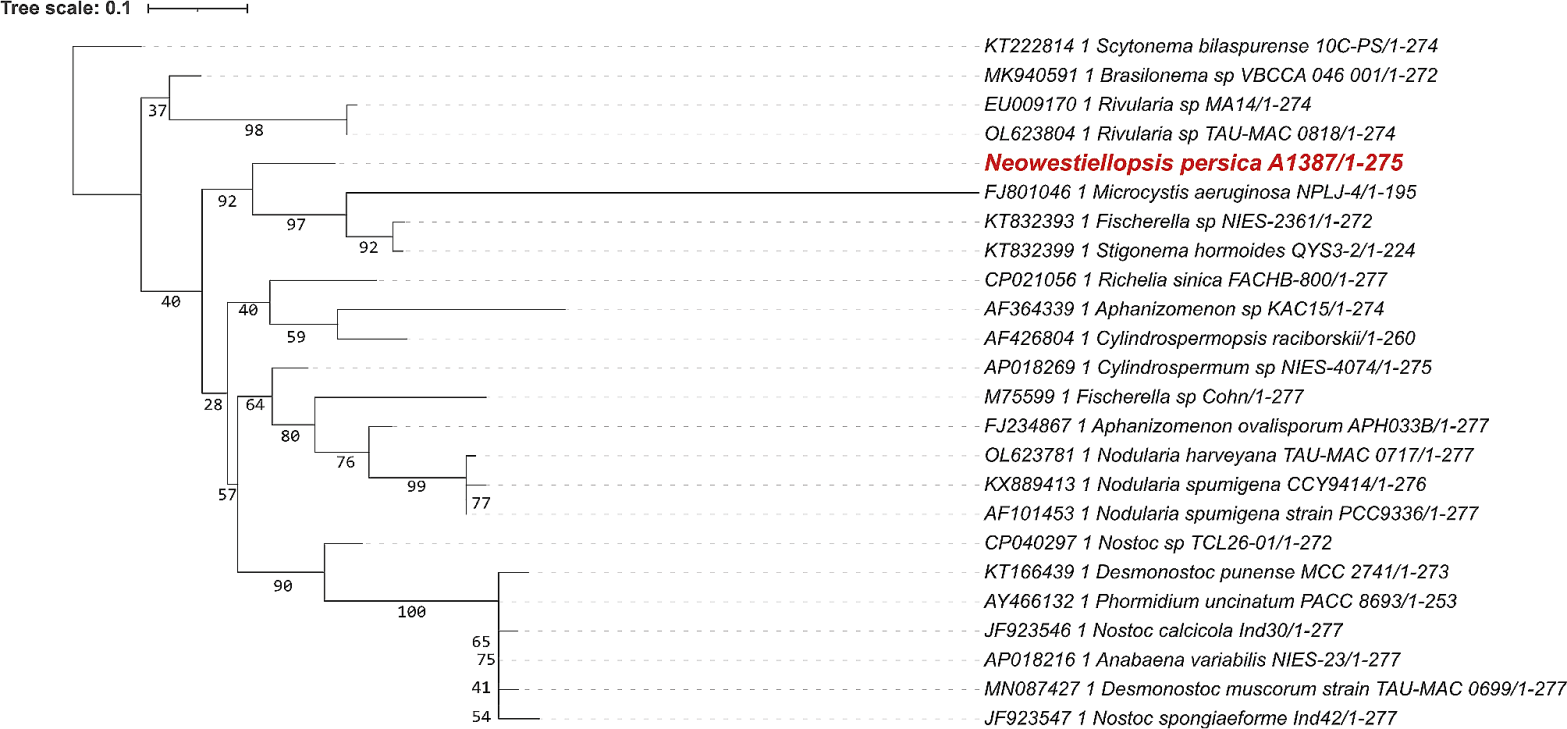
Phylogenetic tree constructed from nucleotide sequences of the *cpcA* gene: Bootstrap values are shown besides each branch, bootstrap values lower than 30 are not shown. The sequence of the studied strain is highlighted in red

**Fig. 3:**
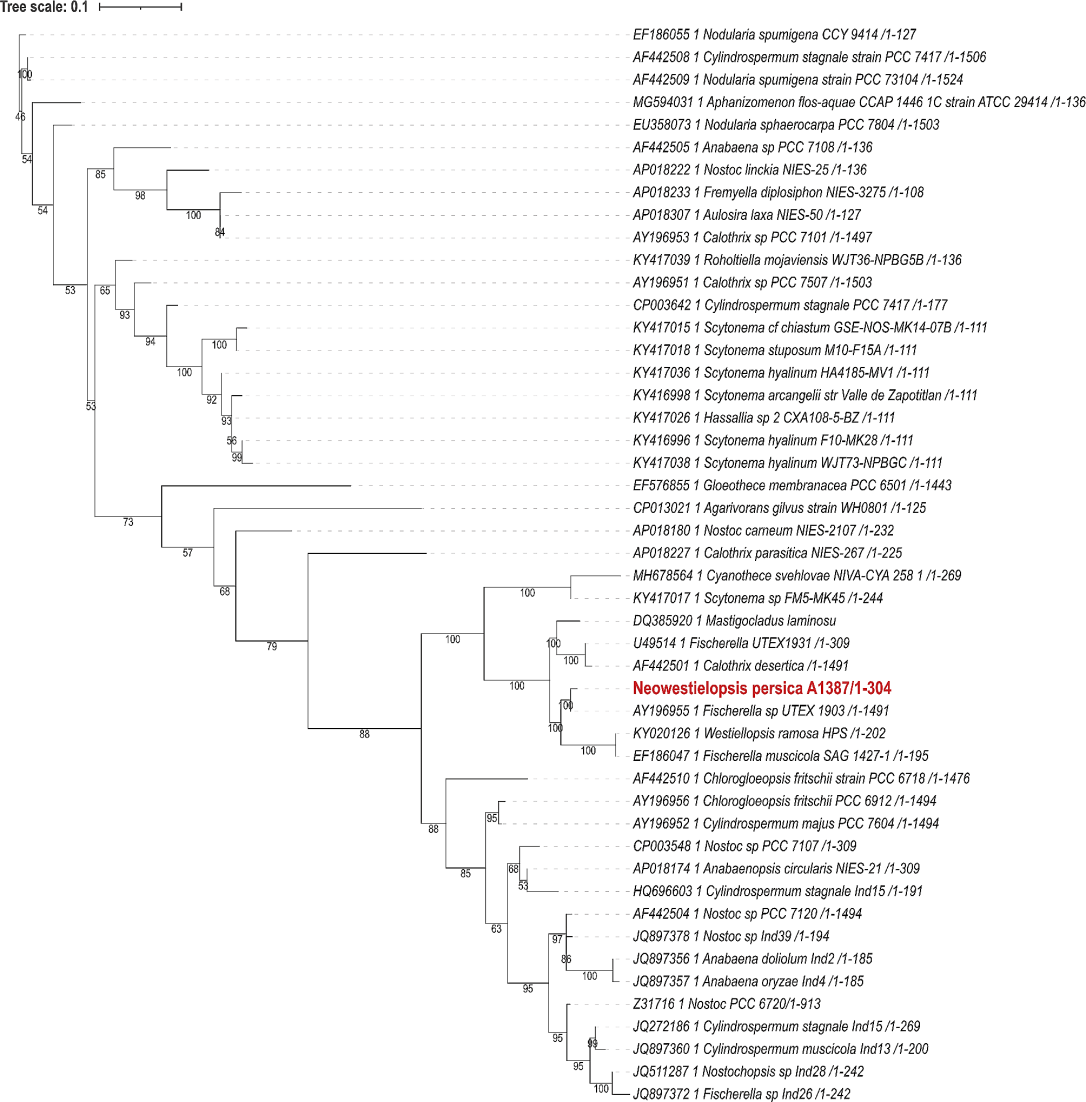
Phylogenetic tree constructed from nucleotide sequences of the nifD gene: Bootstrap values are shown besides each branch, bootstrap values lower than 30 are not shown. The sequence of the studied strain is highlighted in red

**Fig. 4:**
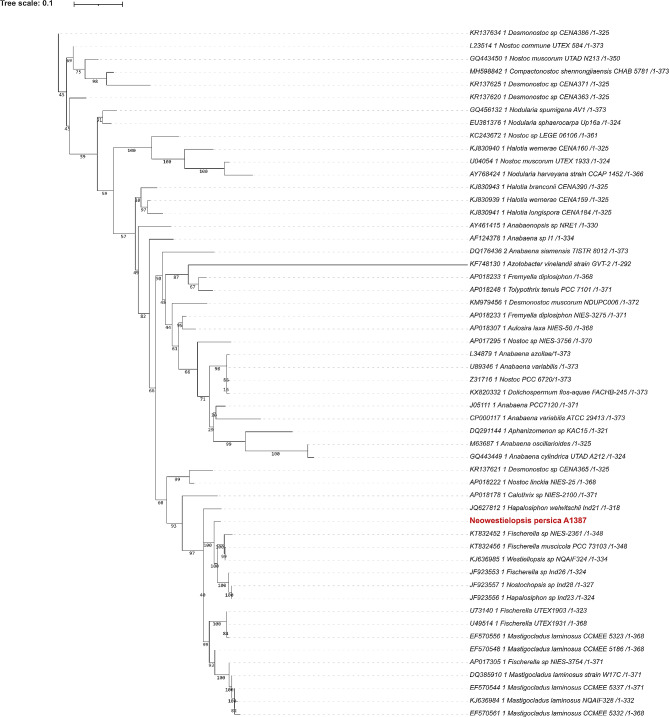
Phylogenetic tree constructed from nucleotide sequences of the *nif*H gene: Bootstrap values are shown besides each branch, bootstrap values lower than 30 are not shown. The sequence of the studied strain is highlighted in red

**Fig. 5:**
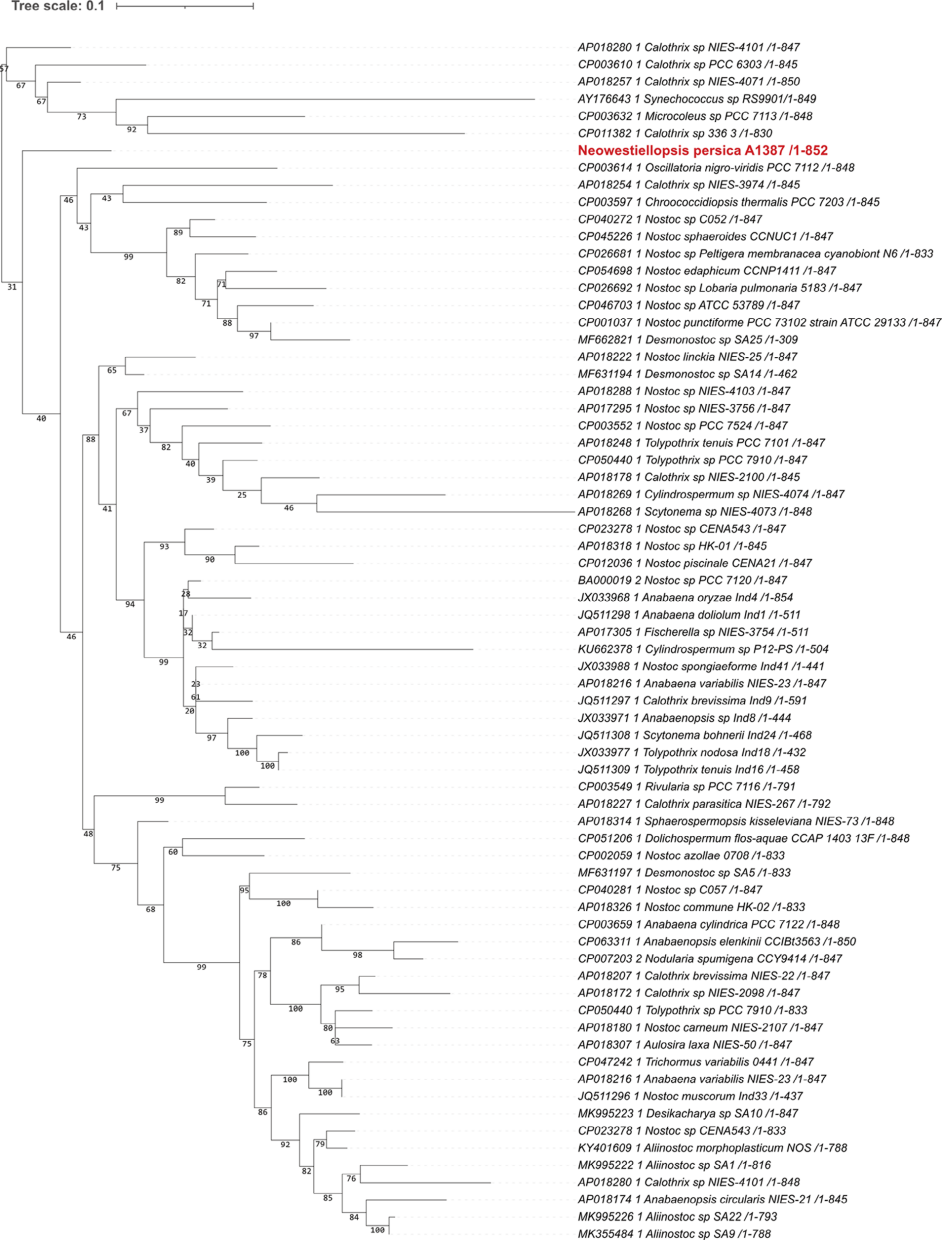
Phylogenetic tree constructed from nucleotide sequences of the *psb*A gene: Bootstrap values are shown besides each branch, bootstrap values lower than 30 are not shown. The sequence of the studied strain is highlighted in red

**Fig. 6:**
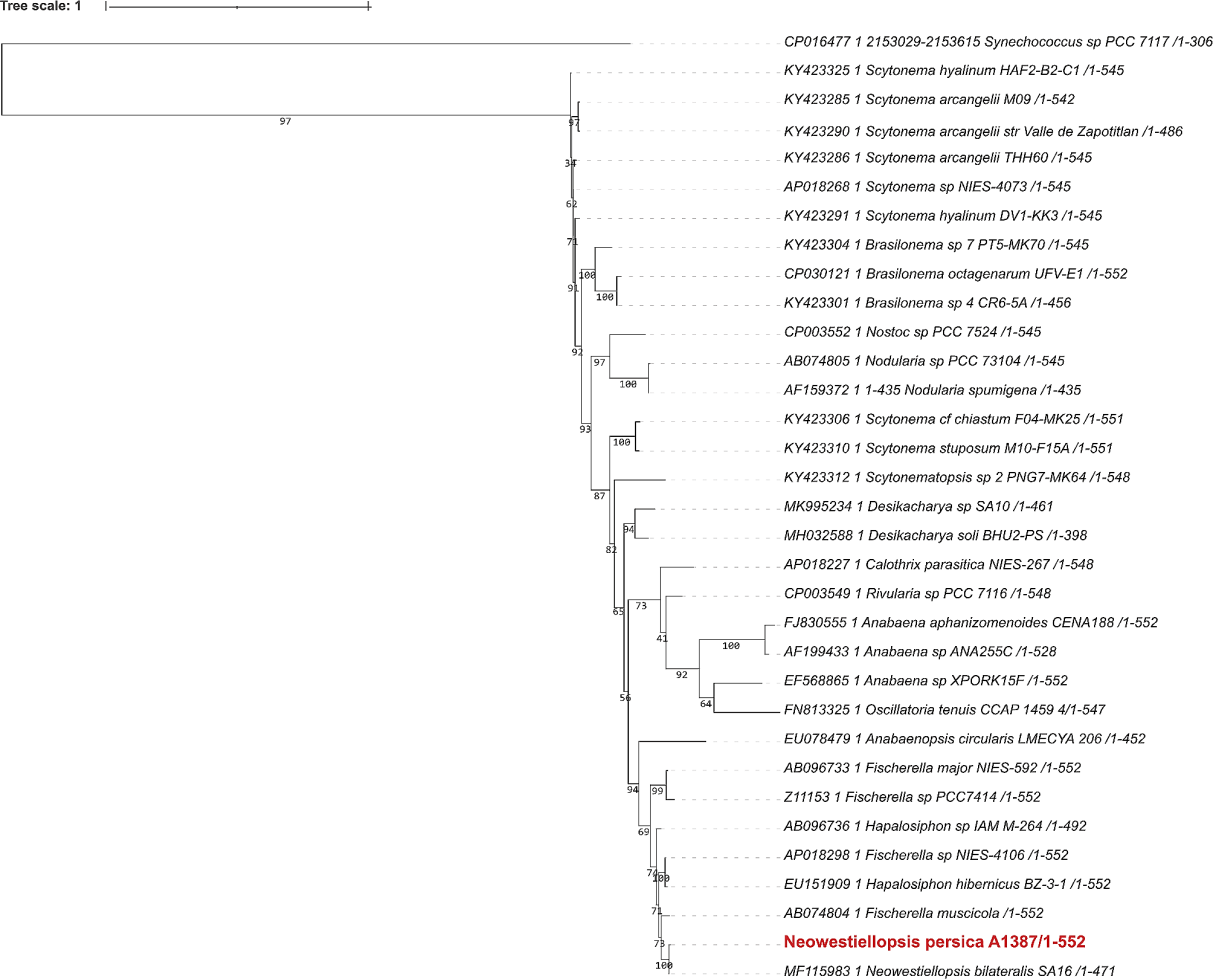
Phylogenetic tree constructed from nucleotide sequences of the*rpo*Cl gene: Bootstrap values are shown besides each branch, bootstrap values lower than 30 are not shown. The sequence of the studied strain is highlighted in red.

These alignments were used for the construction of the phylogenetic trees. For phylogenetic trees, the bootstrap value was set to 1000. Phylogenetic trees based on different gene markers are shown in Figs. 2, 3, 4, 5 and 6, circles indicate standard bootstrap support (%).

In the case of the *cpcA* gene, the trees were built from 24 nucleotide sequences. As shown in Fig. 2, the sequence from the studied strain *Neowestiellopsis persica* A1387 is closely related to the *cpcA* gene from *Fischerella* sp. NIES 2361 (KT832353), *Microcystis aeruginosa* NPLJ 4 (FJ801046) and *Stigonema hormoides* (KT832399| with bootstrap support of 92%.

For the gene *nif*D, 48 nucleotide sequences were used to calculate the phylogenetic tree (Fig. 3). Interestingly, the sequence from the studied *Neowestiellopsis persica* A1387 is placed close to the root of the tree branch. This sequence shows close evolutionary relationships with the *nif*D sequence from *Fischerella and Westiellopsis* species (bootstrap support 100%), namely *Fischerella* sp. UTEX 1903 (AY196955), and *W*. *ramosa* HPS (KY020126) (Fig. 4). From the same family as *nif*D, we also analysed another gene, *nif*H. In this case, we used 54 sequences. As shown in Fig. 4, *nif*H from *Neowestiellopsis persica* A1387 closely clusters with *nif*H sequences from several *Fischerella* strains (JF923553, KT832452, and KT832456) with high bootstrap support (100%).

Another gene we analysed was *psb*A. We built a phylogenetic tree constructed with *psb*A that included 70 cyanobacterial sequences of this gene (Fig. 5). The sequence from *N. persica* A1387 forms a separate branch which clusters with the clade containing *Fischerella* sp. NIES-3753 (AP017305), with a bootstrap value of 40. Other closely related sequences were observed with *Nostoc* species (CP003552), in addition to several species of the genus *Calothrix*, all with high confidence based on the bootstrap values of the branches.

The last of the genes analysed was *rpo*Cl. For the phylogenetic analysis of this gene, we aligned 32 cyanobacterial sequences and then used this alignment to calculate the phylogenetic tree. The *rpo*Cl sequence from *Neowestiellopsis persica* A1387 formed a cluster with two more sequences from the genus *Neowestiellopsis*, namely *Neowestiellopsis persica * SA33 (MF115984), and *Neowestiellopsis bilateralis SA16* (MF115983). Both cases showed strong support with bootstrap values of 100. The cluster contained two other sequences, one from the genus *Fischerella* (AP018298 and AB074804), and one from the genus *Hapalosiphon* (EU151909)(Fig. 6). Using Bayesian inference, trees for each gene were constructed (Supplement Figure S6). This tree supports the position of strains in the tree without interference, with the exception of the psbA gene, which forms a separate branch with strain *Fischerella* sp. NIES-3754 (AP017305) and cpcA, where it belongs to a branch with strain *Fischerella* sp. Cohn (M75599).

### Sequence similarity analyses

The sequences of genes*psb*A, *rpo*C1, *nif*D, *nif*H and *cpc*Afrom *Neowestiellopsis persica* strain A1387 were analysed and their similarity was compared with the sequences sequentially close to them. As our analysis shows, *N. persica* A1387 undoubtedly belongs to the genus *Neowestiellopsis*. The *psb*A gene sequence similarity (Table 3) showed the highest similarity to *Fischerella* sp. NIES 4106 (AP018298), the pairwise similarity being 92.42%. In case of the *rpo*C1 gene (Table 4), the sequence was found to be the most similar (99.41%) to *Neowestiellopsis persica* SA33 (MF115984) and (99.36%) to *Neowestiellopsis bilateralis* SA16 (MF115983). The sequence similarity between *N. persica* SA33 and *N. bilateralis* SA16 was also 99.41%, which suggests, together with the different morphology and ability to produce toxins, that our studied strain could possibly be a different species than *N. persica* SA33. With the strain *Fischerella* sp. NIES-4106 (AP018298), the pairwise similarity in the *rpo*C1 gene was 94.74% and with *Hapalosiphon* sp. IAM_M-264 (EU151909) was 94.71%. The *nif*Dgene sequence similarity (Table 5) was found to be closest to *Fischerella* sp. UTEX_1903 (AY196955) with pairwise similarity being 99.01% and *Fischerella muscicola* SAG_1427-1 (EF186047) with 94.35% similarity. The *nif*H gene (Table 6) showed the closest similarity at 99.1% with *Fischerella* sp. NQAIF311 (KJ636982) and 95.8% similarity with strain *Westiellopsis* sp. NQAIF324 (KJ636985). The biggest differences based on similarity was observed in the *cpc*A gene. The closest strains were *Fischerella* sp. NIES-2361 (KT832393) with 87.13% similarity and *Brasilonema* sp. VBCCA_046_001 (MK94059) with 87.13% similarity (Table 7).

**Table 3:** Pairwise distance matrix (p-distances, %) of the psbA gene (666 bp) for *Neowestiellopsis persica* A1387 and closely related strains

1. OP698108 Neowestiellopsis persica_psba									
**2. AP018298** ***Fischerella*** **sp. NIES-4106**	92.42								
**3. AP017305** ***Fischerella*** **sp. NIES-3754**	91.73	92.66							
**4. AP018268** ***Scytonema*** **sp. NIES-4073**	89.26	90.05	88.31						
**5. AP018174** ***Anabaenopsis circularis*** **NIES-21**	87.84	87.59	88.60	87.84					
**6. CP003552** ***Nostoc*** **sp. PCC_7524**	86.89	87.33	87.83	88.90	89.62				
**7. CP012036** ***Nostoc piscinale*** **CENA21**	86.62	87.10	86.86	88.28	92.40	92.89			
**8. CP003549** ***Rivularia*** **sp. PCC_7116**	86.47	87.73	86.72	89	78.22	87.98	87.35		
**9. AP018314** ***Sphaerospermopsis_kisseleviana*** **NIES-73**	86.32	87.45	87.36	89.38	89.87	91.38	89.23	88.74	
	**1**	**2**	**3**	**4**	**5**	**6**	**7**	**8**	**9**

**Table 4:** Pairwise distance matrix (p-distances, %) of the *rpoC1* gene (591 bp) for *Neowestiellopsis persica* A1387 and closely related strains

1. OP698109 Neowestiellopsis persica rpoC1										
**2. MF115984** ***Neowestiellopsis persica*** **SA33**	99.41									
**3. MF115983** ***Neowestiellopsis bilateralis*** **SA16**	99.36	99.41								
**4. AP018298** ***Fischerella*** **sp. NIES-4106**	94.74	96.47	95.32							
**5. AB096736** ***Hapalosiphon*** **sp. IAM_M-264**	94.71	94.70	94.69	95.12						
**6. EU151909** ***Hapalosiphon hibernicus*** **BZ-3-1**	94.56	96.17	95.11	99.63	95.32					
**7. AB074804** ***Fischerella muscicola***	94.56	95.29	95.96	95.10	95.73	94.92				
**8. AP017305** ***Fischerella*** **sp. NIES-3754**	92.39	92.35	91.93	91.84	91.66	91.66	91.66			
**9. AB096733** ***Fischerella major*** **NIES-592**	92.39	92.35	91.93	91.84	91.66	91.66	91.66	100		
**10. Z11153** ***Fischerella*** **sp. PCC7414**	91.84	92.05	91.50	92.02	91.26	91.84	91.84	96.92	96.92	
	**1**	**2**	**3**	**4**	**5**	**6**	**7**	**8**	**9**	**10**

**Table 5:** Pairwise distance matrix (p-distances, %) of the *nifD* gene (372 bp) for *Neowestiellopsis persica* A1387 and closely related strains

1. OP698110 Neowestielopsis_persica_nifD										
**2. AY196955** ***Fischerella*** **sp. UTEX_1903**	99.01									
**3. EF186047** ***Fischerella_muscicola*** **SAG_1427-1**	94.35	94.35								
**4. AP017305** ***Fischerella*** **sp. NIES-3754**	94.07	95.14	92.82							
**5. DQ385920** ***Mastigocladus laminosus*** **B15A**	94.07	95.14	92.82	100						
**6. KY020126** ***Westiellopsis ramosa*** **HPS**	94.05	94.55	100	93.06	93.06					
**7. U49514** ***Fischerella*** **sp. UTEX1931**	93.75	94.82	92.30	95.14	95.14	92.57				
**8. AY196954** ***Scytonema*** **sp. PCC_7814**	93.75	93.69	92.30	95.14	95.14	92.57	100			
**9. AF442512** ***Fischerella muscicola*** **PCC_7414**	93.75	94.82	92.30	95.14	95.14	92.57	100	100		
**10. AF442511** ***Scytonema hofmanni*** **PCC_7110**	93.75	93.62	92.30	95.14	95.14	92.57	100	99.93	100	
	**1**	**2**	**3**	**4**	**5**	**6**	**7**	**8**	**9**	**10**

**Table 6:** Pairwise distance matrix (p-distances, %) of the *nifH* gene (369 bp) for *Neowestiellopsis persica* A1387 and closely related strains

1. OP698107 Neowestielopsis_persica_nif_H gene										
**2. KJ636982** ***Fischerella*** **sp. NQAIF311**	99.10									
**3. KJ636985** ***Westiellopsis*** **sp. NQAIF324**	95.80	96.40								
**4. KT832455** ***Fischerella muscicola*** **SAG_1427-1**	95.40	96.70	99.40							
**5. JQ627812** ***Hapalosiphon welwitschii*** **_Ind21**	94.65	90.88	91.19	95.28						
**6. KT832451** ***Fischerella*** **_sp. NIES-2361**	94.54	95.80	98.50	98.27	94.65					
**7. U73140** ***Fischerella*** **sp. UTEX1903**	94.42	95.04	94.73	94.11	88.67	93.49				
**8. KT832449** ***Mastigocladus*** **sp. FACHB-785**	94.25	95.80	94.31	94.25	93.71	94.82	96.59			
**9. EF570553** ***Mastigocladus laminosus*** **CCMEE_5201**	92.39	95.50	94.31	93.67	94.65	93.96	96.59	96.83		
**10. U49514** ***Fischerella*** **sp. UTEX1931**	92.11	94.91	94.61	93.67	93.39	92.81	100	95.97	95.65	
	**1**	**2**	**3**	**4**	**5**	**6**	**7**	**8**	**9**	**10**

**Table 7:** Pairwise distance matrix (p-distances, %) of the *cpcA* gene (270 bp) for *Neowestiellopsis persica* A1387 and closely related strains

1. OP698106 Neowestiellopsis persica A1387 cpcA										
**2. KT832393_** ***Fischerella*** **sp. NIES-2361**	87.13									
**3. MK940591** ***Brasilonema*** **sp. VBCCA_046_001**	87.13	85.66								
**4. AP018269** ***Cylindrospermum*** **sp. NIES-4074**	86.54	82.35	84.92							
**5. KT832399 Stigonema hormoides QYS3-2**	86.16	98.66	85.26	83.48						
**6. AY466132** ***Phormidium uncinatum*** **PACC_8693**	85.77	83.79	80.23	88.53	77.23					
**7. AY466120** ***Nostoc linckia*** **PACC_5085**	84	83.08	79.41	87.27	83.48	100				
**8. MN087429** ***Trichormus variabilis*** **TAU-MAC_2510**	84	83.08	79.41	87.27	83.48	100	100			
**9. MN087431** ***Nostoc*** **sp. TAU-MAC_0799**	83.63	82.35	80.88	86.18	82.58	93.28	93.50	93.50		
**10. AY768464** ***Nostoc*** **sp. PCC_7120**	82.90	83.08	78.67	86.18	83.03	98.81	98.91	98.91	92.41	
	**1**	**2**	**3**	**4**	**5**	**6**	**7**	**8**	**9**	**10**

## Discussion

In the present work, we extended our molecular analyses for *Neowestiellopsis persica* strain A1387 by using the *psb*A, *rpo*C1, *nif*D, *nif*H and *cpc*Agenes, with the aim of adding this data to databases for *Neowestiellopsis persica* in order to help with a better understanding of phylogenetic relationships between species belonging to this genus. The only information for any of these studied genes from the genus *Neowestiellopsis* is for the *rpo*C1 gene [23]. Regarding *N*. *persica* SA16 (MF066911) and *N.bilateralis* SA23 (MF066912), the closest similarities were with *Hapalosiphon hibernicus* B2-3-1 (EU151909) and *Fischerella muscicola* (AB075910), with a 96% similarity for both of these strains [23]. For strain A1387, the most similar strains were *N. persica* SA16 (MF066911) with 99.36% similarity and *N.bilateralis* S23 (MF066912) with 99.41% similarity. Other closely related sequences within the clade *Neowestiellopsis* were *Fischerella* sp. NIES 4106 (AP018298) with 94.74% similarity and *Hapalosiphon* sp. IAM-M-264 with 94.71% similarity. For the gene *psb*A the mostsimilar sequence was *Fischerella* sp. NIES-4106 (AP018298). For gene *nif*D, the closest strain was *Fischerella* sp. UTEX 1903 (AY196955) and for *nif*H the closest strain was *Fischerella* sp. NQAIF3111 (KJ636982). Regarding similarity to the *cpc*A gene, the closest strain was *Fischerella* sp. NIES2361 (KT832393) at 87.13%. Usually, phylogenetic trees based on *psb*Aand *nif*D genes have relatively similar characteristics [34], although problems with phylogenetic tree construction could be caused by multiple copies of some genes in genomes. For example, multiple copies of the *psb*Agene can be found in cyanobacteria [35], with nine *psb*Acopies in *Fischerella* sp. PCC9605 encoding the G4-D1 protein. Furthermore, phylogenies based on these genes do not correspond with cyanobacterial phylogenies based on 16 S rRNA [36]. However, if we want to use this gene for characterizing closely related sequences or strains, these sequences always group together within the *psb*A based tree. It seems that closely related strains tend to have similar D1 protein complements [37]. Furthermore, this gene seems to be suitable for use in comparing communities in similar environments, because the number of *psb*A D1 gene copies depend on environmental conditions [38].

The *rpoC1* gene represents genes that are present as single copies in the genome and this molecular marker is usually more discriminatory towards differentiation at the species level than 16 S rRNA [39]. In closely related species, this gene is used for better divergence and for issues at the species level [40]. In heterocytous cyanobacteria, it was usually used for a better understanding of relationships between closely related species within the genera *Minunostoc* [41], *Calothrix, Tolypothrix, Scytonema* [42] or *Anabaena* [43].Usually, the phylogenetic trees constructed based on *rpo*C1 correspond with the phylogeny based on 16 S rRNA. In our case when we compared the phylogenetic tree based on 16 S rRNA [24], *N.* persica A1387 belonged to a well-defined clade with strains *N.persica* SA33 (MF066912), *Neowestiellopsis* sp. KHW5 (MN656995) and *Fischerella* sp. (AJ 544,076), and with the closest clade belonging to *Hapalosiphon* sp. SAG2376 (MK953008) and *Fischerella ambigua* UTEX 1903 (KJ768871). Furthermore, based on the original description of the genus *Neowestiellopsis*, the strains form a clade near the *Pelatocladus* clade formed by strains such as *Hapalosiphon hibernicus* B23-1 (EU151900) and *Pelatocladus maniniholoensis* HA4357-MV3 (JN385293). The other nearest clade, the *Hapalosiphon* clade, is formed by strains such as *Hapalosiphon* sp. Sama 45 (GQ354274), *Westiella intricata* UH HT-29-1 (KJ67016) and *Westiellopsis prolifica* SAG 16.93 (AJ544086) [23]. In the phylogenetic tree, based on *rpo*C1,the topology of clades is similar, and the formed clades correspond with the topology of the phylogenetic tree based on the 16 S rRNA gene. The *nif*H and *nif*D genes are present only in cyanobacteria containing heterocytes and in picocyanobacteria [44]. Furthermore, the operon *nif*HDK is essentially conserved in the genome with minimum translocation and insertions [45].

These molecular markers are usually used in diazotrophic communities and in the past have helped to resolve the phylogeny of closely related species of the genera *Anabaena, Aphanizomenon* and *Nostoc* [46, 47], *Trichodesmium* [48] with the *nif*H gene, and *Nostoc* and *Anabaena* by the *nif*D gene [49].

Although the*cpc*A gene is not suitable for closely related species, it is ideal for multi-locus analyses and identification of strains at the genus level [50]. Based on this marker the genera *Nodularia* [51], *Anabaenopsis* [52], *Aphanizomenon* [53], *Arthrospira* and *Microcystis* [54–57] have been previously studied. Similarly, alignment of *Westiellopsis*sp. Ind19 and *Hapalosiphon welwitschii* Ind21 provided a substantial verification of the placement of monoseriate true branching forms as mentioned by Komárek et al. [58]. However, as of now they have all been placed in the family Hapalosiphonaceae and the use of the phycocyanin locus in this study, supports this placement of the true branching forms. Thus it is evident that the cpc*BA*-IGS locus was robust enough in differentiating the twelve freshwater strains as per taxonomic classification [20]. Our study of this gene shows that *Neowestiellopsis* form a well-established clade.

Based on the 16 S analyses presented by Nowruzi et al. [24], strain A1387 was found to be *N. persica*. However, differences in morphology, production of cyanotoxins, as well as differences in gene sequence similarities for *rpo*C1suggest that this strain could possibly be a different species, or at least a different morphotype of this species. For a better understanding of the phylogeny of *Neowestiellopsis*, more information and sequences, mainly from the genera *Neowestiellopsis, Fischerella* and *Westiellopsis* are needed.

### Electronic supplementary material

Below is the link to the electronic supplementary material.

Supplementary Material 1

Supplementary Material 2

Supplementary Material 3

Supplementary Material 4

Supplementary Material 5

Supplementary Material 6

Supplementary Material 7

## Data Availability

The datasets generated and/or analyzed during the current study are available in the datasets generated and/or analyzed during the current study for 16 S rRNA are available in the [GenBank -] repository, [https://www.ncbi.nlm.nih.gov/nuccore/MZ327713] and accession number as: MZ327713. *nif*D are available in the [GenBank -] repository, [https://www.ncbi.nlm.nih.gov/nuccore/OP698110] and accession number as: OP698110. *nif*H are available in the [GenBank -] repository, [https://www.ncbi.nlm.nih.gov/nuccore/OP698107] and accession number as: OP698107. *psb*A are available in the [GenBank -] repository, [https://www.ncbi.nlm.nih.gov/nuccore/OP698108] and accession number as: OP698108. *rpo*C1 are available in the [GenBank -] repository, [https://www.ncbi.nlm.nih.gov/nuccore/OP698109] and accession number as: OP698109. *cpc*Aare available in the [GenBank -] repository, [https://www.ncbi.nlm.nih.gov/nuccore/OP698106] and accession number as: OP698106.

## Declarations

### Live vertebrates and/or higher invertebrates

This study is not on live vertebrates and/or higher invertebrates.

### Images

All the images are original and not copy from other papers.

## Abbreviations

S: Seconds
min: Minutes
h: hour
*psb*A: gene encoding the PSII reaction center protein D1
*rpo*C1: The chloroplast genes RNA polymerase C1
*cpc*A: phycocyanin intergenic spacer sequences
*nif*H and *nif*D: nitrogen fixation (nif)gene.
