# Supplementary figures and images for: Characterization of Neowestiellopsis persica A1387 (Hapalosiphonaceae) based on the cpcA, psbA, rpoC1, nifH and nifD gene sequences
Source: BMC Ecol Evol. 2024 May 6;24:57. doi: 10.1186/s12862-024-02244-z (PMC11075313; doi:10.1186/s12862-024-02244-z)

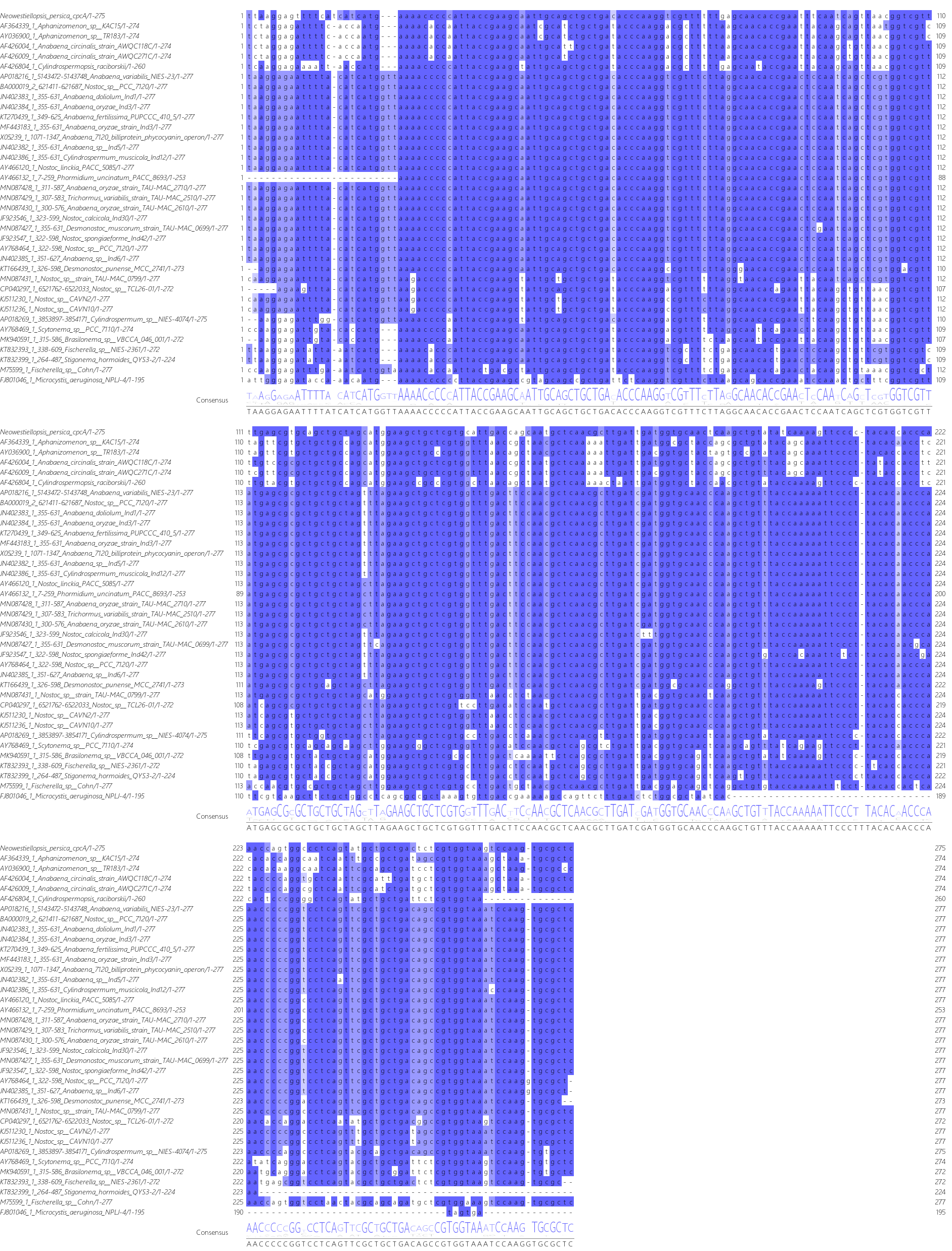

Supplement: Supplementary file 1 — Supplementary Material 1 [file 12862_2024_2244_MOESM1_ESM.png]

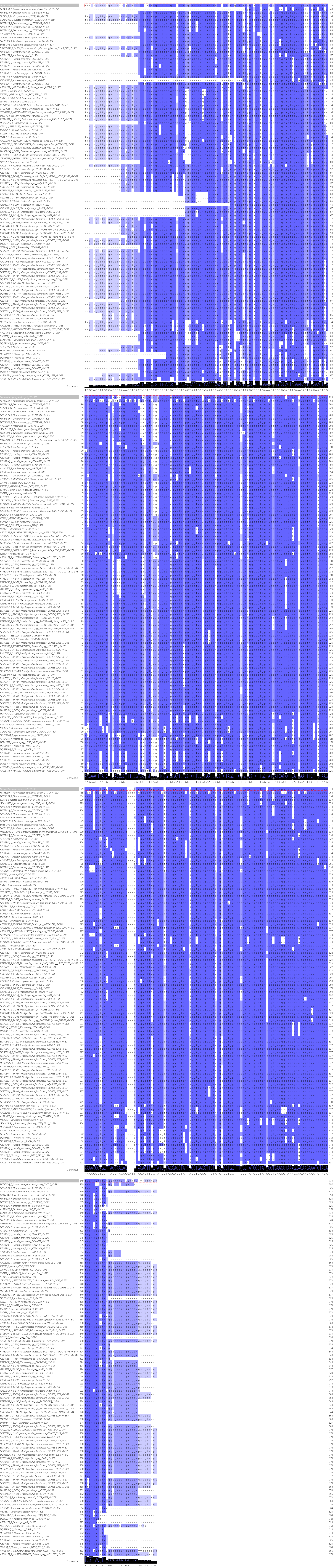

Supplement: Supplementary file 2 — Supplementary Material 2 [file 12862_2024_2244_MOESM2_ESM.png]

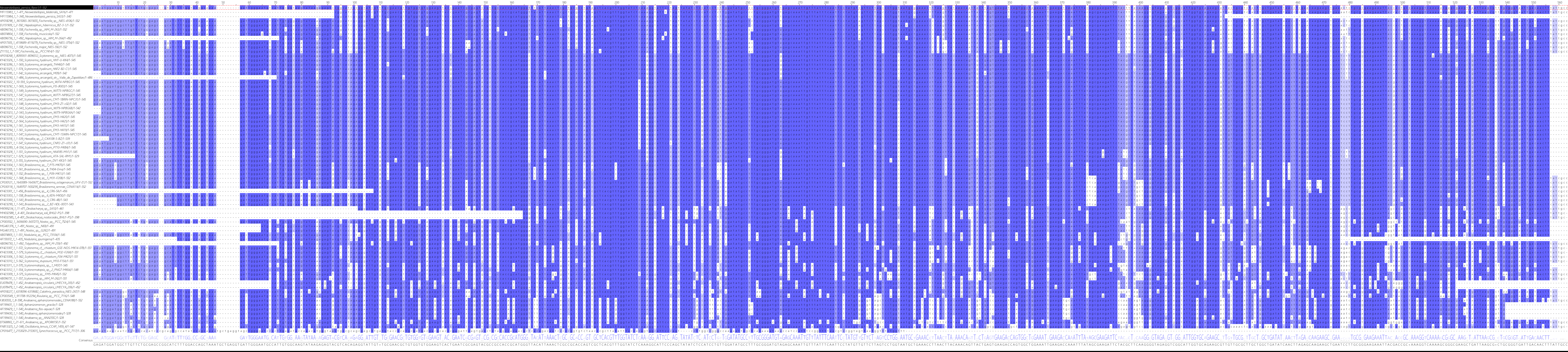

Supplement: Supplementary file 5 — Supplementary Material 5 [file 12862_2024_2244_MOESM5_ESM.png]
