# Supplementary material for: Characterization of Neowestiellopsis persica A1387 (Hapalosiphonaceae) based on the cpcA, psbA, rpoC1, nifH and nifD gene sequences
Source: BMC Ecol Evol. 2024 May 6;24:57. doi: 10.1186/s12862-024-02244-z (PMC11075313; doi:10.1186/s12862-024-02244-z)
Supplement: Supplementary file 6 — Supplementary Material 6 [file 12862_2024_2244_MOESM6_ESM.docx]

**Supplementary figure captions**

Figure S1. Sequences alignments of *cpcA* gene. Similarities in the sequences were highlighted.

Figure S2. Sequences alignments of *nifH* gene. Similarities in the sequences were highlighted.

Figure S3. Sequences alignments of *nifD* gene. Similarities in the sequences were highlighted.

Figure S4. Sequences alignments of *psbA* gene. Similarities in the sequences were highlighted.

Figure S5. Sequences alignments of *rpoCl* gene. Similarities in the sequences were highlighted.

Figure S6. Phylogenetic tree constructed from nucleotide sequences of the studied genes with Bayesian interference: Bootstrap values are shown besides each branch, bootstrap values lower than 30 are not shown.
